# Quantitative light element profiling in plant tissues with monochromatic X-ray fluorescence analysis: a new frontier for abiotic stress studies

**DOI:** 10.1093/jxb/erag291

**Published:** 2026-06-17

**Authors:** Parvinderdeep S Kahlon, Pinelopi Kokkinopoulou, Silvia Bugallo Alfageme, Cheuk Ka Leong, Chenyu Zhang, Zewu Chen, Christa Testerink, Antony van der Ent

**Affiliations:** Laboratory of Plant Physiology, Wageningen University and Research, Wageningen, The Netherlands; Laboratory of Plant Physiology, Wageningen University and Research, Wageningen, The Netherlands; Laboratory of Plant Physiology, Wageningen University and Research, Wageningen, The Netherlands; Laboratory of Plant Physiology, Wageningen University and Research, Wageningen, The Netherlands; Laboratory of Genetics, Wageningen University and Research, Wageningen, The Netherlands; Z-Spec, Albany, NY, USA; Laboratory of Plant Physiology, Wageningen University and Research, Wageningen, The Netherlands; Laboratory of Genetics, Wageningen University and Research, Wageningen, The Netherlands; University College Dublin, Ireland

**Keywords:** Elemental profiling, plant ionomics, plant nutrient uptake, salinity tolerance, X-ray fluorescence

## Abstract

Determining elemental concentrations in plant tissues is essential for physiological studies on abiotic stress. However, high-throughput routine analysis of light elements, ranging from sodium to calcium, in plants is challenging due to the need for complete sample dissolution and expensive and time-consuming inductively coupled plasma-MS (ICP-MS). Ion chromatography and ion-selective electrodes are low-cost methods but suffer from major drawbacks, including limited throughput and time-consuming sample preparation. We report a new methodology for quantitative analysis of light elements in plants using monochromatic X-ray fluorescence (MXRF) analysis. We quantitatively assessed sodium and potassium uptake in *Arabidopsis thaliana*, *Oryza sativa*, and *Lactuca sativa* in salinity treatments. MXRF provides reliable results from samples as small as 1 mg, making it suitable for analysis of seedlings. This is enabled by the high sensitivity of the system and optimized sample preparation that ensure sufficient signal even at low sample masses. We tested the accuracy and precision of the technique for other light elements to demonstrate its broad applicability. The results show that the method delivers rapid, non-destructive, and extraction-free light element analysis on small samples, highly correlating with ICP-MS. MXRF provides accurate measurements and reproducible results for studying salinity tolerance ideally suited for investigating elemental composition during early plant development, offering new possibilities for research into early responses to stimuli.

## Teaser

A rapid MXRF method enables precise, extraction-free analysis of light elements in small plant samples, advancing studies of salinity tolerance and early development.

## Introduction

Plants are essential for sustaining life on Earth. The nutrients they absorb directly determine their ability to grow, thrive, and support other life forms. In plant nutrition, a range of elements can be defined as either essential or beneficial to a varying degree (Brown *et al*., 2022). Out of the 17 essential elements for plant growth, 11 are classified as light elements, ranging from sodium (atomic number, *Z*=11) to calcium (atomic number, *Z*=20). These light elements play a crucial role throughout the life cycle of plants in growth and development, especially nitrogen (N), phosphorus (P), and potassium (K), which are the most common components in commercial fertilizers. Addition of these elements in terrestrial ecosystems has been shown to be beneficial for above-ground biomass (Fang *et al*., 2024). However, in saline soils, the concentrations of elements, such as sodium (Na) and chloride (Cl), are higher, and their uptake by plants leads to toxicity due to competition with beneficial elements (Zhao *et al*., 2020). Depending on the level of salinity in soil, this change of beneficial to disruptive ion uptake, and the decrease in water uptake can lead to severe consequences from defective phenotypes in plants to loss of a whole crop (Zörb *et al*., 2019).

Plants take up elements as ions, and this uptake is tightly regulated by membrane-localized ion transport proteins. Under saline conditions, Na^+^ competes with essential ions such as K^+^ and Ca^2+^, creating an imbalance in physiological functions. To maintain cellular function, plants exclude Na^+^ or transport it into vacuoles, minimizing its toxic effects, and maintain K^+^ uptake to regulate osmotic potential (Munns and Tester, 2008; Van Zelm *et al*., 2020; Barzana *et al*., 2021). Significant efforts have been invested in understanding the mechanisms and role of Na^+^ transporters in enhancing salt tolerance, a critical trait for improving plant performance, a key characteristic important for agricultural production (Zhao *et al*., 2020). In various crops at different developmental stages, quantitative trait loci (QTLs) related to salinity stress have been identified, particularly involving Na^+^ and K^+^ transport genes (Afzal *et al*., 2023). Consequently, one effective approach involves analysing elemental concentrations, such as Na and K, across diverse germplasm to assess the potential for developing new salt-tolerant breeding lines.

To perform elemental analysis, various methods have been developed to date. Conventional methods include inductively coupled plasma-mass spectroscopy (ICP-MS), ion chromatography (IC), and ion-selective electrodes (ISEs), which each have their individual advantages and limitations (Goyal *et al*., 1993; Rieger and Litvin, 1998). ICP-MS offers the highest sensitivity with low limits of detection, but necessitates full acid digestion of the plant material prior to analysis (Masson *et al*., 2010). This is time-consuming, expensive, and often requires a specialized digestion microwave (Djingova *et al*., 2013; Wilschefski and Baxter, 2019). The use of IC and ISE also requires time-consuming sample preparation involving complete solubilization of the plant material, pH adjustment, and ionic strength adjustment (for ISE) (Frenzel and Michalski, 2016). None of these conventional methods is high throughput and they suffer from incomplete dissolution of the sample matrix. This is especially important for light elements, notably silicon (Si) and aluminium (Al) which are notoriously refractory in acid digests, unless hydrofluoric acid digestion is used, which is a highly hazardous laboratory procedure (Taber *et al*., 2002; Arslan and Lowers, 2024).

Furthermore, detection of light elements using extraction-based methods typically requires acid digestion of at least ∼5 mg of dried plant material, followed by highly sensitive analytical techniques such as ICP-MS. However, even under ideal conditions, this introduces a practical limitation. For example, assuming a sodium (Na) concentration of 2500 ppm in plant tissue, and digestion of 5 mg of sample into 10 mL of solution, the resulting analyte concentration is only ∼1.25 mg L^−1^ Na. In studies targeting early developmental stages or small model systems, obtaining sufficient biomass for such extraction-based workflows is challenging. Therefore, non-extraction-based analytical approaches capable of operating on very small sample masses (<10 mg) are highly desirable for salt stress physiology studies. X-ray fluorescence (XRF) spectroscopy does not require solubilization of plant material, and avoiding digestion makes it potentially high throughput (Hossain *et al*., 2021). In combination with simple sample grinding, an XRF instrument can achieve an accuracy comparable with digestion-based methods with ICP atomic emission spectroscopy (ICP-AES), and surpasses the latter for refractory elements, such as Si, because of the aforementioned incomplete solubilization during the digestion process (Reidinger *et al*., 2012; Gazulla *et al*., 2019). An XRF instrument subjects a sample to a beam of X-rays generated by an X-ray tube, inducing X-ray fluorescence in the sample (Jenkins, 1999). The excited fluorescent X-rays are then recorded and analysed to calculate the relative concentrations of elements present in the sample. XRF spectroscopy has low detection limits (<1 mg kg^−1^) for most elements of the Periodic Table, particularly the transition elements. However, the lower the atomic number of the element, the lower the X-ray cross-section (efficiency of XRF generation) and the lower the energy of the fluorescent X-rays. Together this translates to a lower signal produced and to attenuation (absorption) of the emitted fluorescent X-ray in the airpath between the sample and XRF detector. This makes light element analysis with XRF extremely challenging (Marguí *et al*., 2022). In most desktop XRF instrumentats, either vacuum or helium environment is used to reducethe effect of XRF attenuation between sample and detector.

While conventional XRF uses polychromatic excitation, the recently developed monochromatic XRF (MXRF) instrument used in this study (E-lite; https://zspecinc.com) has doubly curved crystals to produce a monochromatic excitation source (Chen and Wittry, 1998). This results in a dramatically lower background with a signal to noise ratio >1:1000, better than polychromatic XRF (Chen *et al*., 2008; Chen and Wei, 2011). In practical terms, this translates to much lower limits of detection, improved spectral deconvolution, and ease of quantification using a Fundamental Parameters approach (Kawai *et al*., 2019). The Z-Spec E-lite instrument incorporates monochromatic X-ray excitation at 4.5 keV (Ti-anode) with polarization and a silicon drift detector with an ultra-thin carbon window to maximize low-energy detection. In a cellulose matrix (wheat flour), Z-Spec Inc. reports limits of detection (LODs) of 3 mg kg^−1^ for Si, 0.5 mg kg^−1^ for S, 0.4 mg kg^−1^ for Cl, 0.3 mg kg^−1^ for K, 0.3 mg kg^−1^ for Ca, and 20 mg kg^−1^ for Al. However, thus far the methodology had not been extended to Na. Given the very low XRF energy (1.040 keV), Na is perhaps the most challenging element to analyse using this method as it is strongly affected by sample and air attenuation of escaping fluorescent X-rays. Yet it is the most crucial element to analyse in NaCl stress studies in plants.

In a previous study, detailed validation of MXRF analysis of plant samples covering light elements (K and Ca), transition metals (Mn, Fe, Co, Ni, Cu, and Zn), metalloids (As and Se), and post-transition ‘heavy’ elements (Tl and Pb) has been reported (Zhang *et al*., 2026). Building on this foundation, the present study develops a method to accurately quantify additional light elements (Mg, Al, Si, P, S, and Cl), including the toxic and beneficial elements Na and K, in plant tissues (roots and shoots) using MXRF technology. In particular, we aimed to test the veracity of this technology for light element analysis (focusing on Na and K) in a range of different samples and plant species; to develop methods for sample preparation and mounting; and to evaluate its accuracy, precision, and repeatability. An additional objective was to test the feasibility of analysing very small sample amounts (<10 mg) generated from experiments involving root systems of young *Arabidopsis thaliana* plants with low biomass. Furthermore, we tested the capacity for analysis of other relevant light elements covering Mg, Al, Si, P, S, Cl, and Ca to demonstrate the capability of the technique beyond salt stress studies, with broader applicability in plant physiology.

## Materials and methods

### *Arabidopsis thaliana* soil experiment

The *Arabidopsis thaliana* Col-0 ecotype plant material was obtained from a salt tolerance assay performed on soil. Seeds were stratified at 4 °C in the dark for 3 d and they were sown on soil in 7×7 cm pots (48 replicates per treatment). The experiment was performed in a climate chamber with 16 h light, 8 h dark, 20 °C/18 °C temperature, 60% humidity, and light intensity of 130 µmol m^−2^ s^−1^ photosynthetically active radiation (PAR). The salt treatment was performed as in Bellinazzo *et al*. (2024) with some modifications. In this experiment, the pots were first watered from the bottom using tap water. Subsequently, 0.5 litres of tap water containing the indicated salt concentration (0, 75, 100, 125, or 150 mM), together with the water remaining from the previous watering step, was added per tray of 24 pots from the bottom. Thereafter, plants were again watered with tap water and Hyponex solution (Supplementary Table S1). Whole rosettes were harvested after 21 d of treatment and dried at 60 °C for 1 week before weighing. Twelve rosettes per treatment were pooled and ground together, constituting a single sample.

### *Oryza sativa* hydroponics assay

*Oryza sativa* seeds (rice) from two varieties (Mudgo and M. Baltec) were obtained from the International Rice Research Institute (IRRI), Philippines. Seeds were dehulled and sterilized with 70% ethanol for 1 min, followed by 30% commercial bleach solution for 25–30 min, then washed with MQ water five times. Germination was conducted using 0.3% Gelrite (Duchefa Biochemie) with Yoshida nutrient solution (Supplementary Table S2; Yoshida *et al*., 1976) in modified PCR tubes, incubated in hydroponic boxes at 37 °C for 24 h and then at 28 °C for 24 h. Plants were grown in a climate chamber at 28 °C/25 °C (day/night) with 75% humidity and 400 µmol m^−2^ s^−1^ PAR. Seedlings which were grown for 14 d in a hydroponic setup using Yoshida nutrient solution (pH 5.2) supplemented with NaCl (0, 25, and 50 mM) were used to test salinity response. The root (washed three times with Milli-Q water) and shoot were harvested and dried at 70 °C for 3 d to determine DWs.

### *Arabidopsis thaliana* plate assay

*Arabidopsis thaliana* Col-0 seeds were surface-sterilized using 70% ethanol for 1 min followed by 10 min of sterilization medium (30% of commercial bleach, 0.02% Triton X-100, Sigma-Aldrich). Then they were sown on 1/2 Murashige and Skoog (MS) medium including vitamins (Duchefa Biochemie), supplemented with 1.1 gL^–1^ MES (Duchefa Biochemie) and 1% Diashin Agar (Duchefa Biochemie), pH 5.8. After 3 d of stratification in the dark at 4 °C, plates with seeds were then placed vertically at 90° in a growth chamber for 7 d in white light (100 µmol m^−2^ s^−1^ PAR, 16 h photoperiod, 20 °C temperature). At day 7, seedlings were transferred to 1/2 MS with or without 125 mM NaCl for the indicated time period. Root samples were collected and rinsed with Milli-Q water three times. Samples were then dried at 60 °C for 1 week.

### *Lactuca sativa* hydroponics assay

Plant material from the *Lactuca sativa* (lettuce) cultivar Cobham green was obtained from a salt tolerance assay performed on hydroponic medium (as described in Ciriello *et al*., 2021; Supplementary Table S3), adjusted to pH 5.8 with KOH. Seeds were stratified for 7 d at 4 °C in the dark, then sterilized with 50% commercial bleach solution containing 0.05% SDS for 5 min and rinsed with sterile Milli-Q water. The seeds were sown on 0.3% Gelrite with the nutrient solution in modified PCR tubes placed in a 0.5 litre hydroponic box with a lid. The boxes were placed in a climate chamber with 16 h light, 8 h dark, 20 °C temperature, 60% humidity, and a light intensity of 140 µmol m^−2^ s^−1^ PAR. On day 3 after sowing, the lid was opened slightly and on day 4 it was fully removed. On day 7, seedlings with open cotyledons and roots protruding from the PCR tube were transferred to 10 litre aerated hydroponic tanks with fresh nutrient medium. On day 9, when seedlings had an established first true leaf, the seedlings were transferred to tanks containing fresh medium and the indicated salt concentration (0 or 100 mM NaCl). On days 12 and 15, seedlings were harvested; three plants were pooled per sample, and three samples were taken per time point and treatment to observe Na uptake and the reduction of K uptake over time. For each sample, the roots, first true leaves, and the remainder of the shoots were harvested and rinsed three times with Milli-Q water, and dried at 60 °C for 3 d, after which they were weighed and ground.

### Elemental analysis of plant samples using monochromatic X-ray fluorescence

Dried root and shoot samples were placed in a 2 mL Eppendorf tube with three metal beads (0.8 mm) or a 12 mL tube with 5–10 metal beads (0.8 mm). The plant material was then ground to a fine powder (<200 µm) in a tissue lyser (Retsch) for 2 mL tubes (oscillation speed of 25 Hz for 120–180 s) and paint shaker (Skandex SK350 Shaker) for 12 mL tubes (180–300 s), and subsamples were inserted into custom XRF sample cups (sample mounting area: 1.35 cm in diameter) and covered with a layer of 4 µm Proline thin film (Chemplex Industries Inc.) for MXRF analysis. The XRF analysis of the plant powdered material was performed using an MXRF instrument (E-lite, Z-Spec Inc.). The instrument uses MXRF excitation at 4.5 keV to analyse elements *Z*=11 (Na) to *Z*=20 (Ca). Samples were analysed for 30–600 s in plant (for samples prepared with the plunger method) or thin sample (for samples prepared with the embedded method) mode. Quality controls including National Institute of Standards and Technology (NIST) Standard Reference Material (SRM) 1515, 1547, 1570a, and 1567b were obtained from the NIST. The detailed protocol is available at Protocols.io (dx.doi.org/10.17504/protocols.io.36wgq1qrkvk5/v1).

### Elemental analysis of plant samples using inductively coupled plasma-mass spectrometry

ICP-MS analysis was performed using dried plant samples ranging from 3 mg to 17 mg. Ion-selective probe measurements were conducted at the Ionomics Facility, University of Nottingham, UK, following the methodology with minor modifications from Danku *et al*. (2013). Samples were digested by adding 1 mL of trace analysis grade (TAG) concentrated HNO_3_ to each tube and pre-digesting overnight in a fume hood, followed by the addition of 0.5 mL of 30% H_2_O_2_ and digestion for 4 h at 115 °C on a block heater. The samples were diluted to 10 mL with Milli-Q water (18 MΩ). Multi-element analysis was carried out using ICP-MS (Thermo Fisher Scientific iCAP-Q, Bremen, Germany) with a flow rate of 1.2 mL min^−1^ via an autosampler (Cetac ASX-520) and an ASXpress™ rapid uptake module, using a perfluoroalkoxy (PFA) Microflow PFA-ST nebulizer. Data were processed with Qtegra™ software, applying external cross-calibration between pulse-counting and analogue detector modes. The system employed in-sample switching between two modes: (i) helium gas with kinetic energy discrimination (KED) to remove polyatomic interferences and (ii) hydrogen gas for selenium determination. Internal standards (Sc and Ge at 10 µg L^−1^, Rh and Ir at 5 µg L^−1^) were introduced via the ASXpress at an equal flow rate to correct for instrumental drift. Calibration included (i) a multi-element standard (Ag, Al, As, Ba, Be, Cd, Ca, Co, Cr, Cs, Cu, Fe, K, Li, Mg, Mn, Mo, Na, Ni, P, Pb, Rb, S, Se, Sr, Ti, Tl, U, V, and Zn) at 0–100 µg L^−1^ (Claritas-PPT grade CLMS-2, SPEX Certiprep, Metuchen, NJ, USA), (ii) a multi-element calibration solution (PlasmaCAL, SCP Science, France) with Ca, Mg, Na, and K at 0–30 mg L^−1^, and (iii) an in-house-prepared mixed phosphorus, boron, and sulfur standard (KH_2_PO_4_, K_2_SO_4_, H_3_BO_3_). Certified reference material NIST 1573a (Tomato Leaves) was digested alongside samples and blanks for quality control.

### Determination of the limits of detection of different elements

To ascertain the LODs of Na, Mg, Al, Si, P, S, Cl, K, and Ca using the MXRF approach, we determined the lower limit of detection (LLD) following Kadachi and Al-Eshaikh (2012). The LLD is the smallest amount of an element in a sample (AR-grade cellulose) that can be detected with a 95% confidence level based on the lowest net peak intensity of an element in the XRF spectrum. The lowest intensity is equivalent to two standard counting errors σB of a set of measurements of the background intensity I_b_ under the element peak of blank samples (cellulose). The LLD is expressed by the following equation (Kadachi and Al-Eshaikh (2012):


(1) $$\mathrm{LLD}=\frac{2\times \sqrt{2}}{\mathrm{IS}}.\sigma_{B}\approx \frac{3}{\mathrm{IS}}\sqrt{\frac{I_{b}}{T_{b}}}$$

For this purpose, we determined the background count in cellulose, and the instrumental sensitivity (IS) in Equation 1, which is the signal counts per ppm determined from the spike at 10 000 mg kg^–1^ (prepared as described below) for the different elements to determine the sensitivity with a 60 s measurement time. This does not take into account that the presence of Cl raises the background of Na due to the Cl escape peak near the Na peak.

In addition, we made calibration curves to assess linearity for the same elements spiked in cellulose using 400 mg of cellulose powder for each element and then spiked with different concentrations (100, 1000, and 10 000 mg kg^–1^) of individual elements using sodium carbonate or sodium chloride (for Na), magnesium acetate tetrahydrate (for Mg), aluminium chloride hexahydrate (for Al), potassium silicate (for Si), ammonium dihydrogen phosphate (for P), ammonium sulfate (for S), manganese chloride tetrahydrate or sodium chloride (for Cl), potassium carbonate (for K), and calcium nitrate (for Ca). The desired concentration of the element was dissolved in 1–4 mL of Milli-Q and water was added to cellulose powder. After mixing thoroughly, samples were dried in a hot air oven at 70 °C for 24 h. The dried cellulose powder was mixed thoroughly and 10 mg was weighed for the embedded method or 300 mg for the plunger method. Each concentration was prepared and measured in triplicate.

### Elemental analysis of Na-, Cl-, and K-spiked cellulose using ‘conventional’ X-ray fluorescence

To compare the LLD of Na, Cl, and K with conventional XRF and MXRF, we performed WD-XRF and MXRF analysis on cellulose powder spiked with NaCl and KCl at different concentrations (0, 1, 10, 100, 1000, 10 000, and 100 000 mg kg^–1^) in triplicate. The conventional XRF used a wavelength dispersive (WD-XRF) instrument (Jaguar S6, Bruker) which has a 400 W source with up to 50 kV and three analyser crystals. A full analysis takes 20 min per sample. Powdered samples were weighed (0.75–1.3 g) in sample cups (Ø 20 mm) and covered with Proline foil (4 µm thickness). The analysis was performed with the SMART-QUANT standardless method included in the Bruker software. Semi-quantitative results were calculated with cellulose as the sample matrix. The MXRF measurements used 10 mg of plant material and a 60 s. measurement time. The cellulose powders spiked with NaCl and KCl were also analysed in air with a custom isotope-based XRF instrument with Fe-55 source (Nečemer *et al*., 2008) and with a home-made handheld system (PDZ-01, JSI) with a Rh anode (35 kV, 10 μA, irradiated area of sample ∼1 cm^2^, distance between detector and sample 23 mm). In both systems, the fitting of the spectra was performed by AXIL (Vekemans *et al*., 1994), while quantitative analysis was performed by software developed by Kump (2024). The quantitative analysis is based on an emission-transmission method, where absorption of a metal target (Mo-Kα or Cu-Kα) is measured on a pellet to determine the dark matrix (C, O, and N content).

### Statistical analyses

Data analyses in this study were performed in R software (version 4.2.0, R Core Team, 2023). A *P-*value of <0.05 was considered statistically significant. The normality of the data for each treatment group was assessed using the Shapiro–Wilk test. If the data followed a normal distribution, an ANOVA was performed to compare the groups at a 95% confidence level. In cases where significant differences were detected, a post-hoc Tukey test, TukeyHSD, was performed. However, if the data did not meet the normality assumption, the non-parametric Kruskal–Wallis test was applied, followed by Dunn’s test for post-hoc comparisons. Pearson’s correlation analysis was carried out to test the correlation of data, and all the measurements were treated and presented individually in the correlations. Plots were created with the R package ggplot2.

## Results

### Development of the new monochromatic X-ray fluorescence methodology for light element analysis

**Fig. 1.**
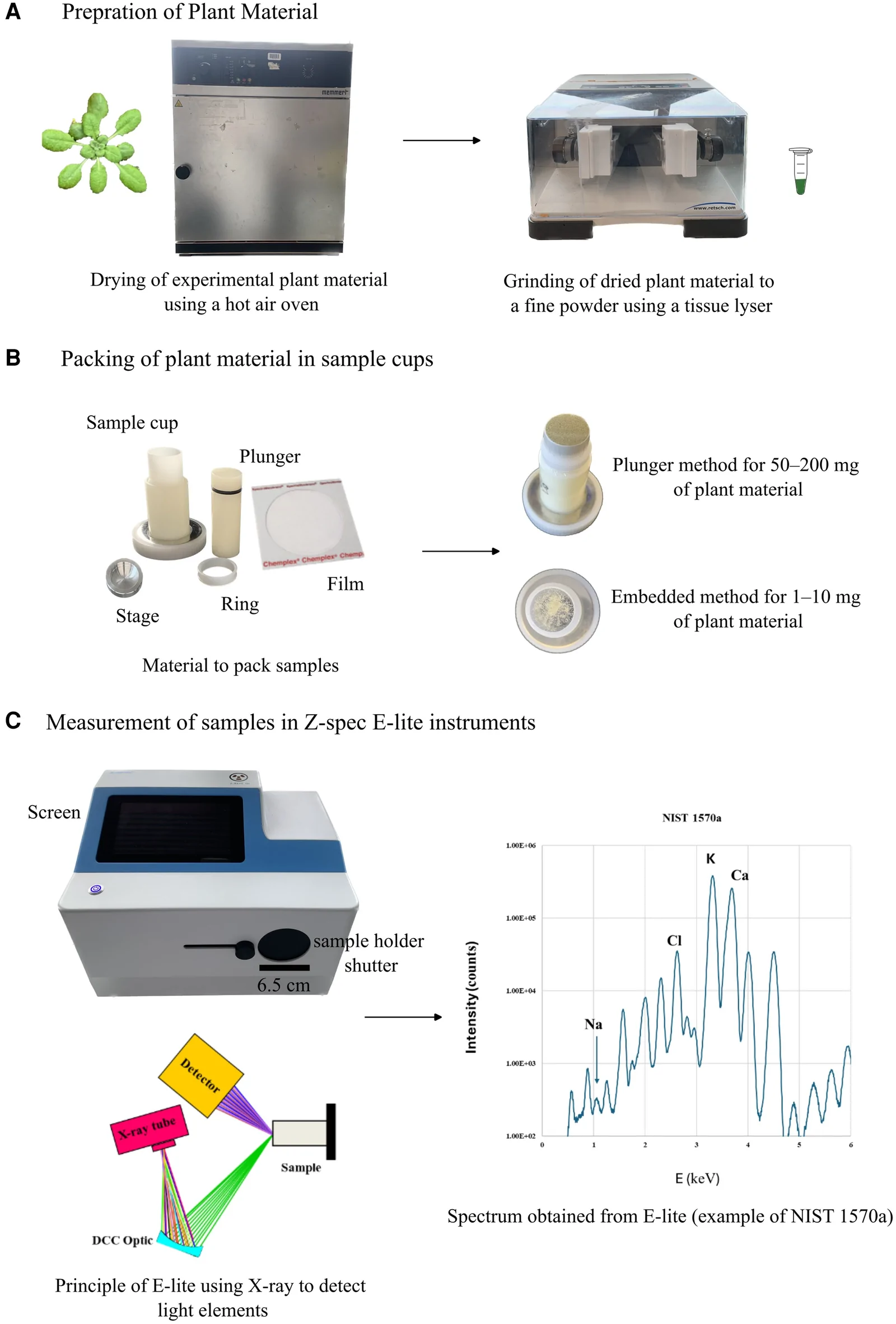
Schematic representation of the experimental procedure utilized for detecting low-energy elements using the Z-Spec MXRF E-lite instrument. The flowchart outlines the key steps involved in the analysis (A) including sample preparation and (B) packing configurations used for dried plant samples. The plunger method compacts plant material directly into sample cups and is typically used for larger sample masses (5–200 mg; ∼200 mg shown here), whereas the embedded method packs smaller amounts of plant material within a matrix between films (1–50 mg; ∼1 mg shown here), enabling measurements with minimal sample material. (C) MXRF instrument, X-ray excitation, and the resulting data acquisition. The output spectrum is derived from the certified reference material NIST 1570a, highlighting the fluorescence peaks of Na, Cl, K, and Ca.

**Fig. 2.**
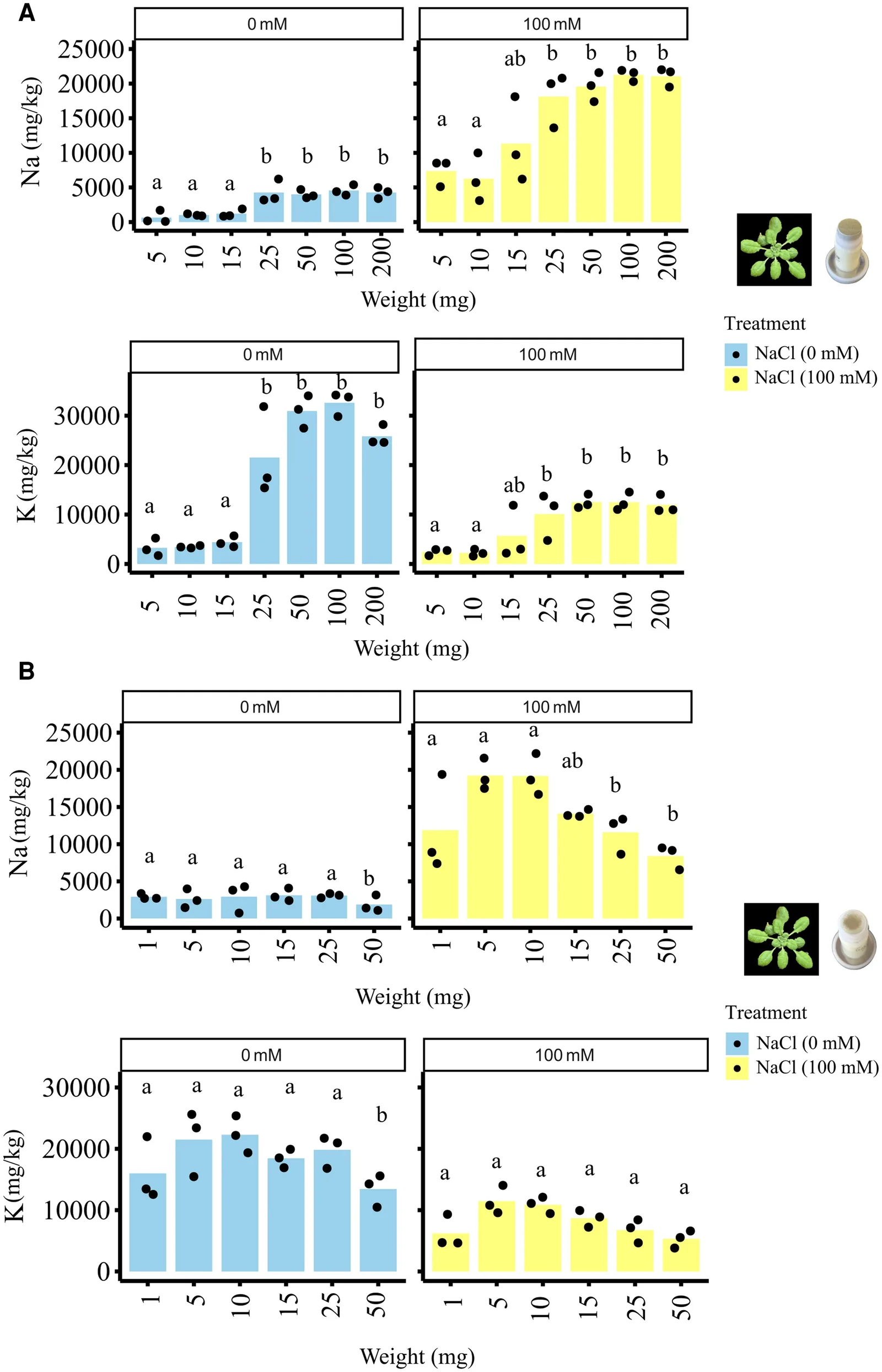
Amount of plant material required for Na and K analysis using two methods. (A) Plunger method (5–200 mg; 60 s) and (B) embedded method (1–50 mg; 60 s). Sodium (Na) and potassium (K) levels are shown in mg kg^–1^ in *Arabidopsis thaliana* rosettes grown under control soil (blue bars) and 100 mM NaCl treatment (yellow bars). The amounts of plant material required for each method are compared across three independent biological replicates (dots). Data were analysed using the non-parametric Kruskal–Wallis test followed by Dunn’s post-hoc test; different letters indicate significant differences (*P*<0.05).

We first tested the amount of biological material required to measure Na and K precisely. We developed and tested two methods. The plunger method, which compacts the sample into sample cups for XRF analysis (see Fig. 1), allows for larger sample sizes (5–200 mg) to accommodate more abundant tissues (Fig. 2A). In contrast, the embedded method, where samples are packed between films (1–50 mg), enables precise measurements with minimal material (Fig. 2B), making it ideal for early developmental stages or scarce samples. Three independent biological replicates were analysed for each condition. The results showed that for the plunger method, 25 mg of material was sufficient to detect Na and K in *A. thaliana* shoot samples, although 25 mg showed higher variance compared with 50, 100, or 200 mg. For the embedded method, 1–10 mg showed the best outcome, with the 1 mg sample having a higher variance compared with 5 mg or 10 mg, which showed more consistent measurements.

**Fig. 3.**
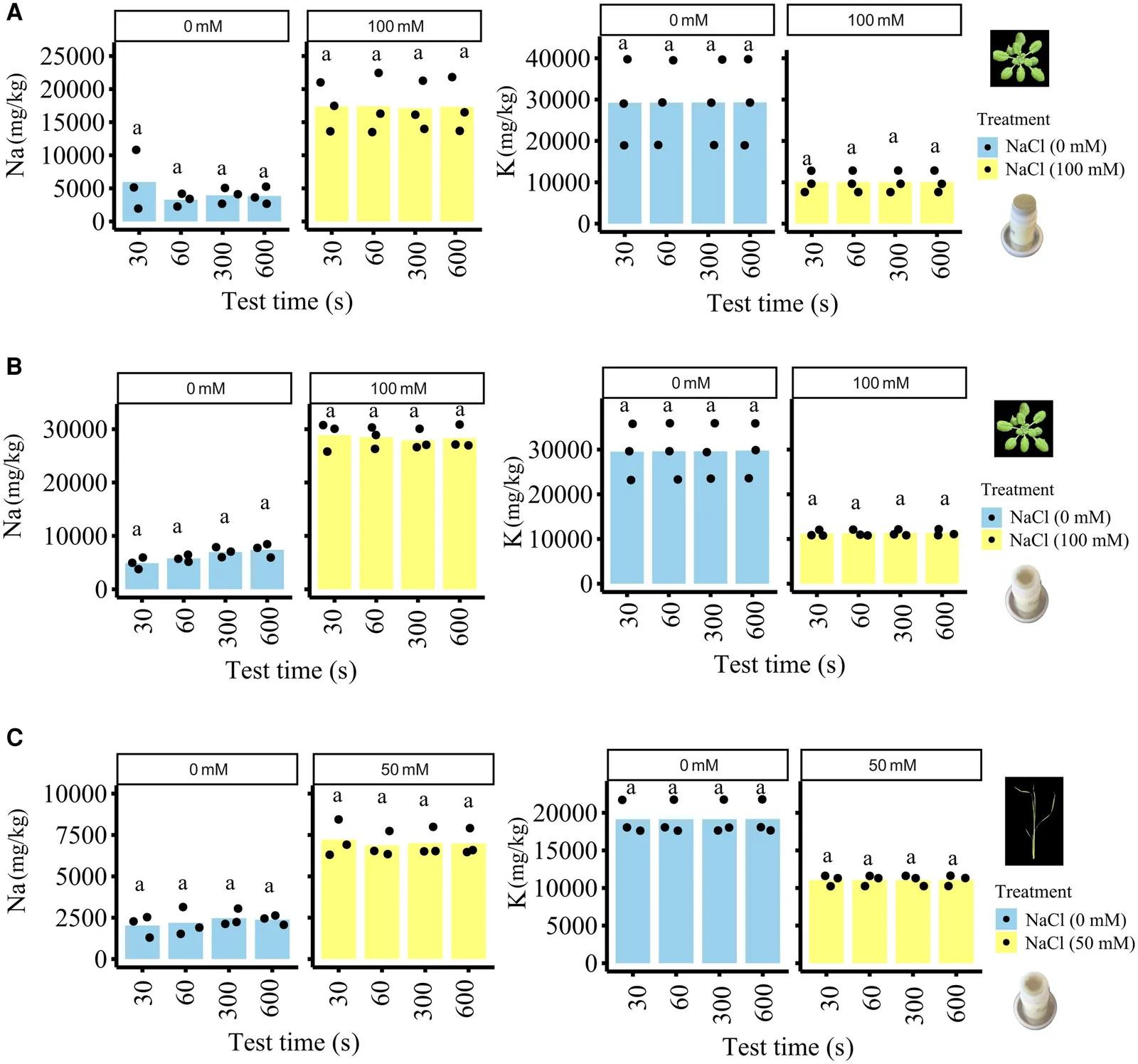
Time of measurement for sodium (Na) and potassium (K) analysis using two methods. (A) Plunger method (200 mg of plant material) and (B and C) embedded method (5 mg of plant material). The graph shows measurement duration for each technique (30–600 s). Measurements were performed on *Arabidopsis thaliana* shoots grown under control soil conditions (blue bars) and soil watered with 100 mM NaCl (yellow bars) for (A) and (B), and on *Oryza sativa* shoots grown hydroponically under control conditions (blue bars) and with 50 mM NaCl (yellow bars) for (C). Differences in measurement time were assessed using three independent biological replicates (each dot represents one replicate). Data were analysed using the non-parametric Kruskal–Wallis test followed by Dunn’s post-hoc test; different letters indicate significant differences (*P*<0.05).

Time of measurement was also tested for *A. thaliana* and *O. sativa* shoot samples (Fig. 3). Test times of 30, 60, 300, and 600 s were used for both the plunger and the embedded method. For each condition, three independent biological replicates were measured. Our data showed no significant differences in the tested times using the plunger method for soil-grown *A. thaliana* under control or 100 mM NaCl conditions (shoot samples; Fig. 3A). Similar to the plunger method, we did not observe any differences in test time for the embedded method (sample preparation with 5 mg of plant material) for the same *A. thaliana* shoot samples (Fig. 3B) and hydroponically grown *O. sativa* under control conditions or supplemented with 50 mM NaCl (shoot samples; Fig. 3C). Thus, our results indicate that neither the plunger nor the embedded method is sensitive to the duration of measurement within the tested time ranges for both *A. thaliana* and *O. sativa* shoot samples. Thus, the measurement time can be chosen based on practical considerations without affecting signal consistency and measurement precision of Na and K detection. Data presented in Figs 2 and 3 evaluated method performance within XRF, specifically the effects of measurement time and sample weight on signal variability and consistency. These experiments were designed to determine optimal measurement conditions.

We then tested the repeatability of this method by using NIST standards 1547 (peach leaves), 1570a (spinach leaves), and 1515 (apple leaves) representing different levels of Na and K. The amounts of 1 mg and 10 mg were tested in embedded form (Table 1), while 250 mg samples (Table 2) were packed using a plunger method. Ten measurements were performed for each sample with 60 s measurement time. The results showed that Na and K levels could be detected in samples as small as 1 mg, while the standard error was lower in the higher weight samples, indicating improved measurement precision.

**Table 1.** Embedded method for testing repeatability of the XRF E-lite instrument using NIST standards 1547, 1570a, and 1515

Element name NIST sample (weights)	Sodium (Na) mg kg^–1^	Potassium (K) mg kg^–1^
1547 (1 mg) ±SE	n.d.	25 700±29.4
1570a (1 mg) ±SE	18 000±312	29 400±189
1515 (1 mg) ±SE	n.d.	16 000±12.8
1547 (10 mg) ±SE	n.d.	29 000±23.9
1570a (10 mg) ±SE	18 900±247	27 800±21.9
1515 (10 mg) ±SE	n.d.	18 300±10.7

Each sample was measured 10 times for 60 s and the average of 10 samples is represented with the SE.

The values of NIST 1547 for Na* is 23.8±1.6 mg kg^–1^ and for K is 24 330±380 mg kg^–1^; NIST 1570a for Na is 18 210±230 mg kg^–1^ and for K is 29 000±260 mg kg^–1^; NIST 1515 for Na* is 0 mg kg^–1^ and for K is 16 080±210 mg kg^–1^. n.d. is not detected. * denotes non-certified values.

**Table 2.** The plunger method with 250 mg of NIST standards 1547, 1570a, and 1515 was performed to test repeatability of the E-lite instrument

Element name NIST sample (weights)	Sodium (Na) mg kg^–1^	Potassium (K) mg kg^–1^
1547 (250 mg) ±SE	n.d.	26 600±29.9
1570a (250 mg) ±SE	18 100±170	31 200±19.0
1515 (250 mg) ±SE	n.d.	18 700±11.0

Each sample was measured 10 times for 60 s, and the average of 10 samples is represented with the SE.

The values of NIST 1547 for Na* is 23.8±1.6 mg kg^–1^ and for K is 24 330±380 mg kg^–1^; NIST 1570a for Na is 18 210±230 mg kg^–1^ and for K is 29 000±260 mg kg^–1^; NIST 1515 for Na* is 0 mg kg^–1^ and for K is 16 080±210 mg kg^–1^. n.d. is not detected. * denotes non-certified values.

### Comparison with other method (ion-selective probe using inductively coupled plasma-mass spectrometry)

**Fig. 4.**
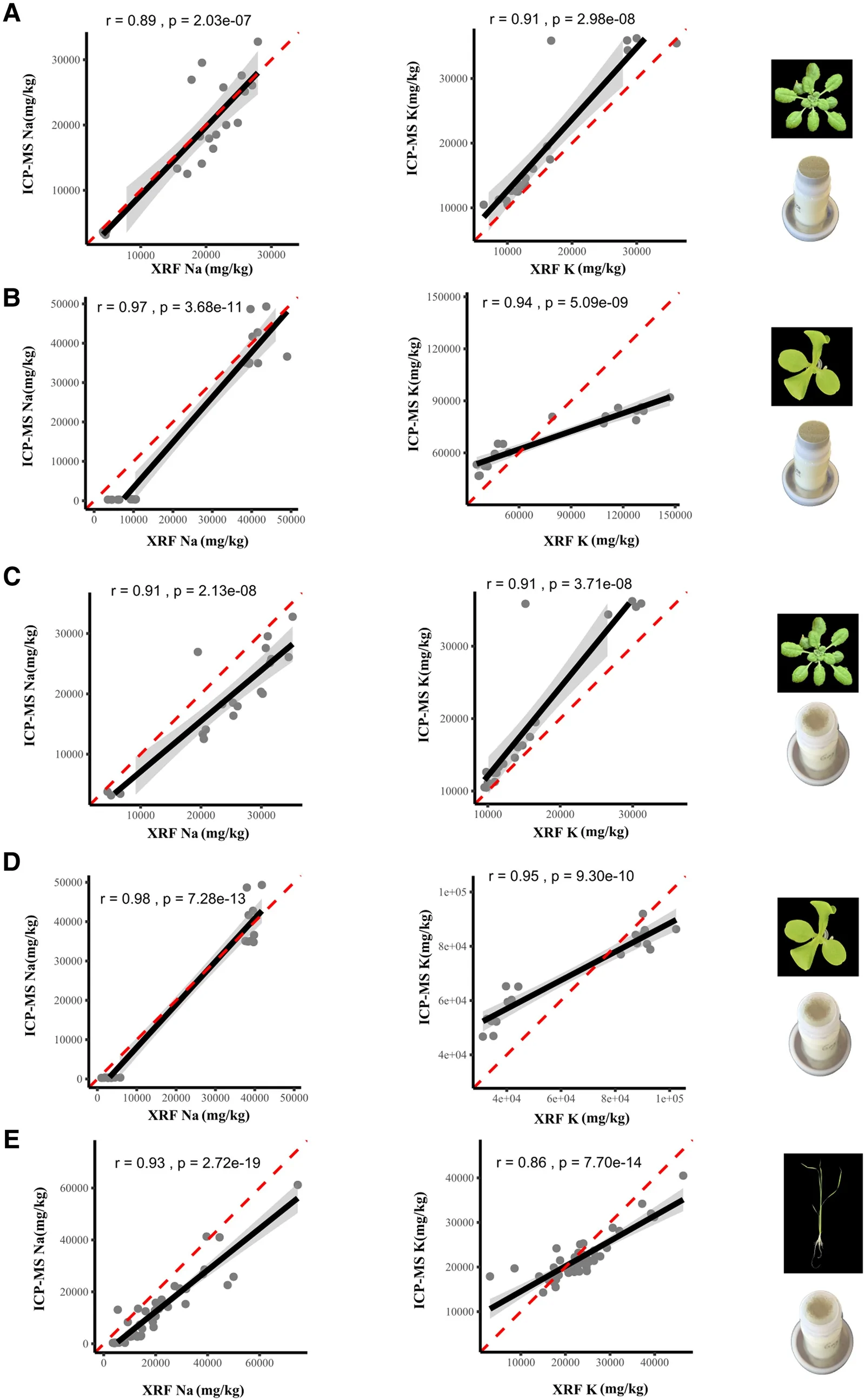
Comparison of measurement techniques for sodium (Na) and potassium (K) analysis using monochromatic XRF and ICP-MS. (A, B) Plunger method; (C–E) embedded method. (A) *Arabidopsis thaliana* shoots treated with 0, 75, 100, 125, and 150 mM NaCl analysed using the XRF plunger method (200 mg, 60 s) and compared with ICP-MS (10–17 mg). (B) *Lactuca sativa* cultivars Salinas and Cobham shoots treated with 0 and 100 mM NaCl analysed using the XRF plunger method (200 mg, 60 s) and compared with ICP-MS (10–17 mg). (C) *Arabidopsis thaliana* shoots treated with 0, 75, 100, 125, and 150 mM NaCl analysed using the XRF embedded method (5 mg, 60 s) and compared with ICP-MS (10–17 mg). (D) *Lactuca sativa* cultivars Salinas and Cobham shoots treated with 0 and 100 mM NaCl analysed using the XRF embedded method (5 mg, 60 s) and compared with ICP-MS (10–17 mg). (E) Shoots and roots of two *Oryza sativa* varieties (Mudgo and M. Blatec) treated with 0, 25, and 50 mM NaCl analysed using the XRF embedded method (5 mg, 60 s) and compared with ICP-MS (3–10 mg). Different NaCl treatments were analysed in three independent biological replicates, with each dot representing an individual sample measurement. Pearson correlation coefficients (*r*) are indicated.

Next, we compared data on Na and K obtained from the MXRF instrument with data obtained from a well-developed ion-selective probe method using ICP-MS (Fig. 4). The dataset included *A. thaliana* shoots grown in soil supplemented with 0, 75, 100, 125, or 150 mM NaCl solution, *O. sativa* seedlings from Mudgo and M. Baltec (shoots and roots) grown on hydroponics solution with 0, 25, or 50 mM NaCl, and *L. sativa* cv Salinas and cv Cobham green (shoots and roots) grown on hydroponics solution with 0 or 100 mM NaCl. Correlation plots were generated on data from *A. thaliana* and *L. sativa* XRF measurements using the plunger method with 200 mg and 60 s measurement and corresponding ICP-MS data from the same samples (Fig. 4A, B), MXRF measurements using the embedded method (5 mg and 60 s measurement time) with the corresponding ICP-MS data from selected samples (Fig. 4C, D), and *O. sativa* samples using the embedded method (5 mg) with the corresponding ICP-MS data from same samples (Fig. 4E). All comparisons were performed using data from individual samples per replicate per treatment. All the data showed significant correlations, with *r* values ranging from 0.86 to 0.98; raw data are depicted in Supplementary Table S4.

### Evaluation of lower limit of detection for different elements using monochromatic X-ray fluorescence

**Fig. 5.**
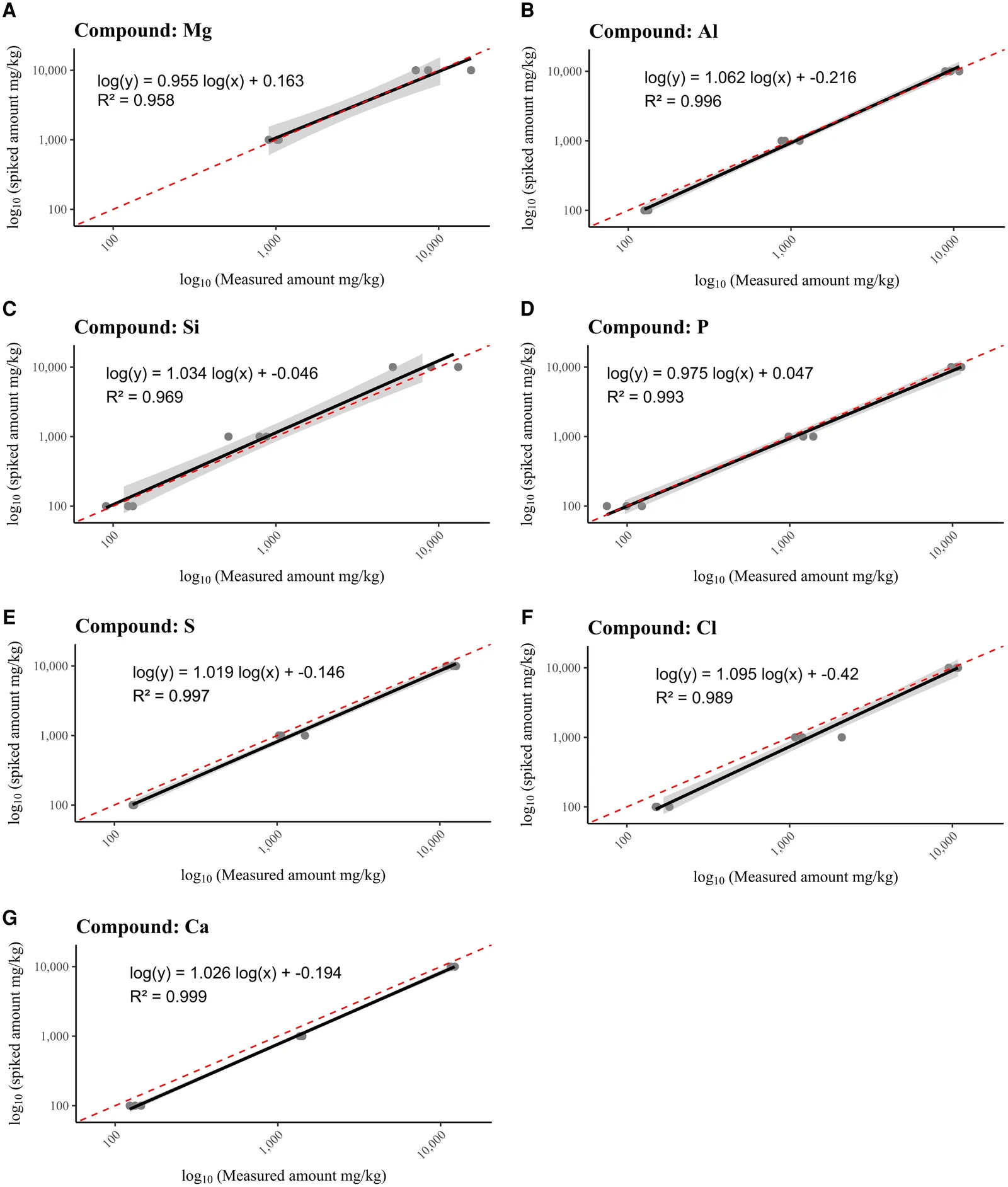
Measurement for Mg, Al, Si, P, S, Cl, and Ca spiking with different concentrations (100, 1000, and 10 000 mg kg^–1^) using the embedded method (10 mg) with a measurement time of 60 s in triplicate. Data points represent measurements from individual spiked samples. Both axes are presented on a log_10_ scale, and Pearson correlation coefficients (*r*) are calculated using log_10_-transformed data. Samples with readout values below the instrument detection limit were not displayed on the log_10_-scaled figure.

We performed spiking experiment for K to evaluate the LLD for the plunger and embedded methods using 60 s and 300 s measurement time wth MXRF (Supplementary Fig. S1). Measured concentrations matched the spiked values across 100–100 000 mg kg^–1^ with both the plunger and embedded methods, and calibration curves showed linearity across this range. For Na, we used only the embedded method with 60 s and 300 s measurement times (Supplementary Fig. S2), and measured concentrations matched spiked values for 1000–100 000 mg kg^–1^ at both measurement times, with linear calibration observed. We also performed a spiking experiment for Mg, Al, Si, P, S, Cl, and Ca to validate the embedded method using 60 s measurement time. The performance of the method in detecting these elements showed significant positive correlations between known element concentrations and measured concentrations in the range of 100–10 000 mg kg^–1^ (Fig. 5).

The LLD was tested for Na, Mg, Al, Si, P, S, Cl, K, and Ca by spiking into cellulose to assess the detection performance of the MXRF analysis. For setting up a reliable spiking method, a detailed testing on K spiking with potassium carbonate was performed (Supplementary Fig. S1). Based on these K spiking data, no obvious differences were observed in either of the tested methods (plunger and embedded method) and at either time (60 s and 300 s). As a result of this, the embedded method was used for spiking of all other compounds. Sodium spiking was performed with two different salts (sodium carbonate and sodium chloride) to estimate the effect of the Cl escape peak. Na spiking with sodium carbonate measurements revealed no obvious differences in outcome based on the time of measurements (Supplementary Fig. S2). Na spiking with NaCl revealed overestimation of Na amounts in our measurements due to the escape peak of Cl (Supplementary Table S5). The data were relative to Na_2_CO_3,_ but one should be aware that in plant samples treated with NaCl the Cl escape peak can cause an overestimation of the Na values. Hence, if the data show prevailing Na and Cl concentrations exceeding 5000 mg kg^–1^ for both elements, then it is advised to post-process the data, rather than to rely solely on the automated Fundamental Parameters algorithm in the instrument. This entails performing a manual fit of the sample spectrum for deconvolution of the Na K-line using the supplied desktop software.

For Mg, Al, Si, P, S, Cl, and Ca spiking, the embedded method was used and 60 s measurement time with 100, 1000, and 100 000 mg kg^–1^ spiking of individual elements was employed. Mg spiking was performed with magnesium acetate tetrahydrate, Al with aluminium chloride, Si with potassium silicate, P with ammonium dihydrogen phosphate, S with ammonium sulfate, Cl with manganese chloride, and Ca with calcium nitrate. The recovery rate of Mg, Al, Si, and P was lower when compared with S, Cl, and Ca. The finding suggests that lighter elements than S have lower recovery, but all the compounds showed significant correlation to the spiked amount and detected amounts (Fig. 5A–G), also evident from the calculated LOD of these compounds (Supplementary Table S6). We then performed a comparison of the newly developed MXRF method with more widely available WD-XRF, as well as isotope XRF and polychromatic XRF using NaCl- and KCl-spiked cellulose powder to assess performance relative to existing XRF technology. The data from MXRF showed better accuracy with the known Na and Cl concentrations values when compared with data obtained from WD-XRF analysis, whereas K concentrations were better measured with other types of XRF compared with MXRF (Supplementary Tables S7, S8). In the case of Na detection, MXRF has a better sensitivity and starts to detect Na at ∼100 mg kg^–1^ whereas WD-XRF detects Na from 10 000 mg kg^–1^ in the cellulose matrix, and Na could not be detected with isotope XRF and polychromatic XRF.

### Applications of the new monochromatic X-ray fluorescence method in plant stress studies

**Fig. 6.**
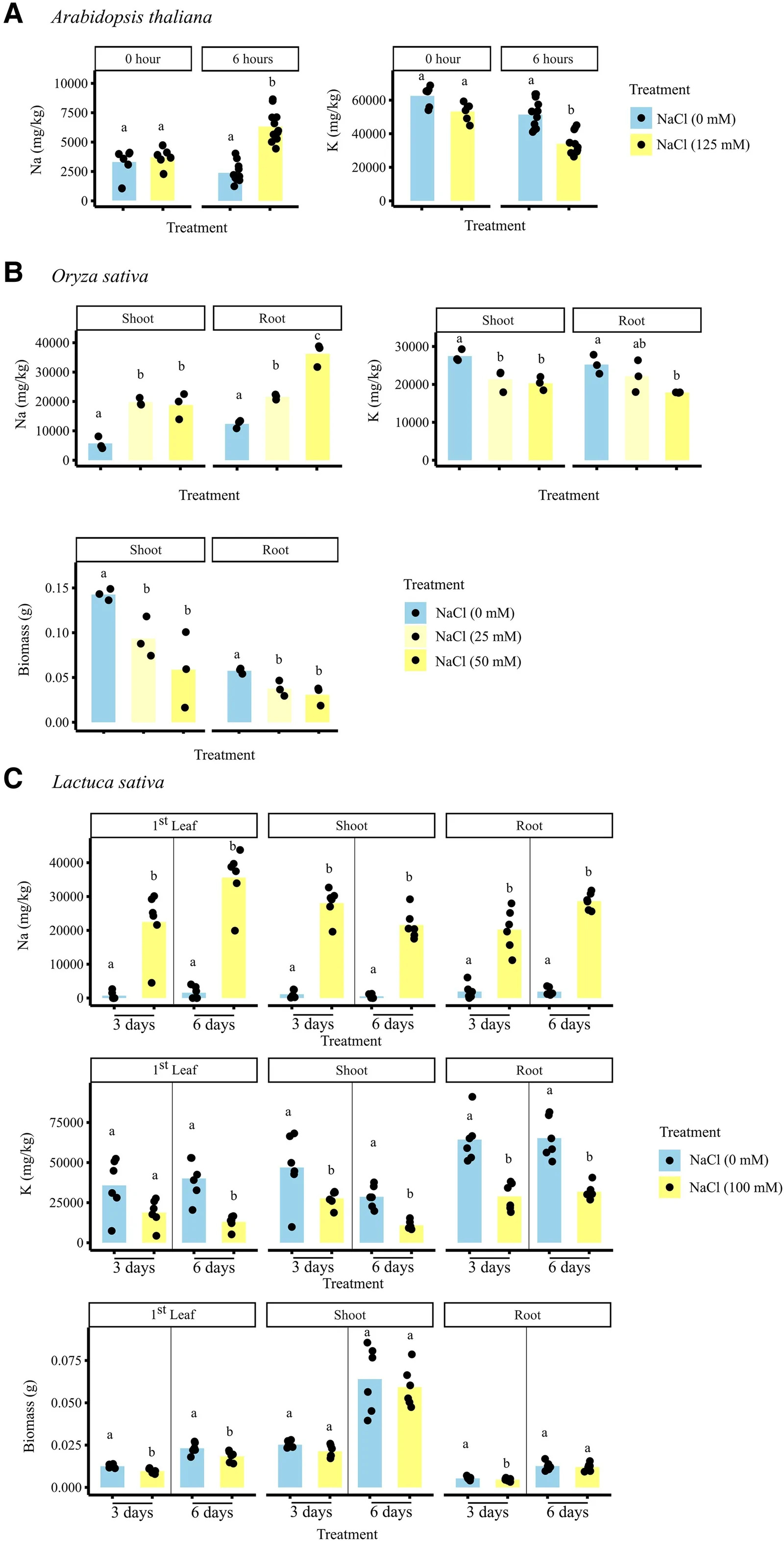
Proof-of-concept results demonstrating the effects of salinity on sodium (Na) and potassium (K) levels using the embedded method with 5 mg sample mass and 60 s measurement time. (A) *Arabidopsis thaliana* (Col-0) seedlings grown on 1/2 MS agar plates and treated with 125 mM NaCl for 6 h; roots were analyzed for Na and K levels. (B) *Oryza sativa* (Mudgo) seedlings grown hydroponically for 14 d and treated with 25 mM or 50 mM NaCl; shoots and roots were analysed for Na and K levels. (C) *Lactuca sativa* (Cobham green) seedlings grown hydroponically and treated with 0 or 100 mM NaCl; roots, first true leaves, and remaining shoots were harvested on days 12 and 15 and analysed for Na and K accumulation and biomass. Blue bars indicate control conditions and yellow bars indicate salt treatments. Each data point represents an independent biological replicate, and different letters indicate statistically significant differences (*P*<0.05). Statistical analyses were performed according to data distribution: normally distributed datasets (*A. thaliana* K; *O. sativa* biomass, Na, and K) were analysed using one-way ANOVA followed by Tukey’s HSD post-hoc test, whereas non-normally distributed datasets (*A. thaliana* Na; *L. sativa* biomass, Na, and K) were analysed using the Kruskal–Wallis test followed by Dunn’s post-hoc test.

We performed a proof-of-concept experiment using roots of *A. thaliana* Col-0 seedlings, which were grown on agar plates for 7 d before being treated with 125 mM NaCl for 6 h. This treatment allowed us to assess the effects of salt on root ion levels by comparing the Na and K levels with those of corresponding controls, including 7-day-old seedlings shifted to NaCl plates and harvested immediately, 0 h samples, and 7-day-old seedlings that were on control medium plates for the 6 h period (Fig. 6A). The data obtained from the root tissues indicated a significant increase in Na concentration and a corresponding decrease in K levels following the 6 h NaCl treatment. This shift in ion balance suggests that the plant is responding to the saline environment by accumulating Na at this early time point.

To further explore the effects of salinity on plant performance, we conducted a salinity experiment with the Mudgo variety of *O. sativa*. The plants were grown in a hydroponic system, with some groups receiving a control nutrient solution and others supplemented with either 25 mM or 50 mM NaCl for 14 d (Fig. 6B). The results from this experiment showed a significant increase in Na levels in both NaCl treatments. Simultaneously, we observed a decrease in K concentrations, which was accompanied by a reduction in both shoot and root dry biomass. To obtain further insight into the effect of salinity on plant performance over time, we conducted a salinity experiment with *L. sativa* cv Cobham green. Plants were grown on a hydroponic system and treated with 0 or 100 mM NaCl (Fig. 6C). The first true leaf, the rest of the shoot, and root samples were harvested to observe the Na and K concentration in the organs over time. The results from this experiment showed a significant increase of Na concentration in all harvested organs at 3 d and 6 d of treatment, as well as a K decrease in all organs at 6 d of treatment.

Overall, our proof-of-concept experiments offer valuable opportunities for the use of *A. thaliana* roots at an early developmental stage where plant material is limited. Furthermore, we show a correlation between mild salinity responses and dry biomass in both *O. sativa* shoots and roots, and find a significant increase of Na and decrease of K in *L. sativa* grown under salt stress.

## Discussion

The results of this study demonstrate that the MXRF method is highly effective for quantifying Na and K concentrations in small amount of plant samples, providing a reliable, non-extraction alternative to traditional extraction-based techniques including ICP-MS. XRF analysis has been shown to be a powerful tool for the analysis of various elements in biological and environmental samples due to its high precision, non-destructive nature, and ability to measure low concentrations of elements (Marguí *et al*., 2005; Castillo-Michel *et al*., 2017). XRF analysis has been successfully used in the past for performing multi-elemental analysis and has shown promising outcomes with both Na and K, but required larger plant samples, up to ∼2 g of plant material (Marguí *et al*., 2005). In the current method, the ability to analyse as little as 1–10 mg of plant material is primarily attributed to advancements in (polarized) MXRF technology itself, which offers enhanced sensitivity and precision compared with polychromatic XRF that is commonly used. Instrumental advancements incorporated in the E-lite analyser include very short sample to detector geometry, the newest generation of low-energy optimized silicon drift detector with a graphene window, and ultra-low noise electronics. Moreover, the use of an innovative method for sample mounting using an approach involving a thin sample layer in a film sandwich ensures a very low scattering background, further improving the signal to noise ratio. This was further improved by utilizing a background subtraction and Compton Scatter correction in the Fundamental Parameters calculation for each sample, and these corrections are now automated in data output. Another important aspect of XRF analysis is sample preparation, including the type, amount, and thickness of the sample. Various preparation methods can be used, such as pressed pellets and loose powders, with smaller particle size to enhance analytical accuracy but all requiring larger amounts of samples (Marguí *et al*., 2022). In contrast, we present a method that requires only 1–10 mg of plant material, embedded between two films. This approach addresses previous limitations by enabling precise measurements with minimal sample sizes, thus facilitating the analysis of scarce biological materials.

The study focuses on light elements, mainly Na and K, which are analysed in roots and shoots of *A. thaliana*, *O. sativa*, and *L. sativa*. We performed a spiking experiment for Na and K to evaluate the lower limit of detection of this method (Supplementary Figs S1, S2). We observed accurate measurements for K 100–100 000 mg kg^–1^, and for Na the measurable range was >1000–100 000 mg kg^–1^. We also performed a spiking experiment for Mg, Al, Si, P, S, Cl, and Ca to validate the performance of this method in detecting these elements with significant linearity and positive correlations of known spiked concentrations and measured concentrations ranging between 100 mg kg^–1^ and 10 000 mg kg^–1^ (Fig. 5). Yet the method has wider potential applications for a variety of tissues from flowers to seeds. In addition, soil and liquid samples can also be tested and analysed to assess nutrient use efficiency comprehensively. Moreover, the ability of the method to provide simultaneous multi-elemental analysis makes it particularly valuable for understanding nutrient uptake dynamics over time, especially when analysing liquid samples. Validation of results obtained through MXRF has been shown to correlate well with established techniques such as ICP-MS, underscoring the accuracy and reliability of this approach (Singh *et al*., 2022).

The MXRF method reported here is a new, accurate, and reproducible approach for the routine analysis of light elements such as Na and K in plant tissues. The contrasting accumulation of Na and reduction of K observed across *A. thaliana*, *O. sativa*, and *L. sativa* upon NaCl treatment (Fig. 6) in different tissues reflects disruption of ion homeostasis, which is a hallmark of salinity stress responses. The ability of the MXRF method to detect these changes in both roots and shoots, including very small *A. thaliana* root samples, demonstrates its suitability for monitoring ion concentrations during early salt sensing and stress adaptation. These findings further support the application of this approach for screening salt tolerance in diverse plant systems. The embedded method developed in this study was recently used by Lamers *et al*. (2025) to detect the content of Na in *A. thaliana* biosynthesis and signalling mutants of the phytohormone abscisic acid (ABA). In that study, the positive role of ABA in regulating ion homeostasis in shoots upon salt stress was reported. Such alterations in early ion homeostasis are critical to understanding the mechanisms plants use to cope with salt stress. The MXRF method offers significant advantages, including the ability to analyse small sample sizes with minimal preparation and no hazardous chemicals, along with improved user-friendliness, improved sensitivity, and reduced analysis time. The sensitivity for Na detection in plant samples using MXRF was significantly better as compared with ‘conventional’ XRF technology. This makes it particularly attractive for applications ranging from breeding programmes to ecological research. By eliminating the need for chemical digestion and allowing for the analysis of very small samples, the method is highly suitable for investigating plant responses to stress, nutrient management, and salinity tolerance, with potential applications in agriculture, plant breeding, and environmental science. Overall, despite some challenges in sample preparation, the MXRF technique represents a promising tool for future research in plant light elemental analysis and will be highly suited for investigating other elements of importance in physiology such as Mg, Al, Si, P, S, Cl, and Ca.

### Limitations of the new monochromatic X-ray fluorescence method

While the MXRF method has demonstrated versatility and reliability, there are some inherent limitations that must be addressed for optimal performance. One notable limitation is the vulnerability of the detector to dust contamination that can affect the uniformity of results, necessitating regular cleaning to maintain accuracy and to prevent contamination. This requirement can add to the maintenance workload and can influence the consistency of results if not properly managed. Additionally, the method requires the use of highly uniform and finely ground dry powder (particles finer than 0.1 mm) from the sample material, as particle size has been shown to have a significant effect on the accuracy of the measurements using XRF analysis (Beckhoff *et al*., 2006; Ben Amar *et al*., 2022). Achieving this level of uniformity can be challenging, especially for different types of plant tissues such as roots, shoots, leaves, and seeds. Any inconsistency in particle size may lead to variability in the results, thus emphasizing the importance of meticulous sample preparation.

Additionally, in energy dispersive XRF analysis, Na peaks can be affected by the escape peaks of Cl (Supplementary Table S5). The escape peak is an artefact resulting from the energy of the photon not being entirely deposited in the Si drifted detector but escaping through the emergence of Si characteristic photons from the detector. The escape peak of the Cl K-beta peak has an energy of 1.086 keV, which is very close to 1.041 keV of the Na K-alpha line. With the detector energy resolution being ∼90 eV at this energy, this peak overlap can be effectively handled through peak deconvolution, provided the Cl peak is not overwhelming.

## Supplementary Material

erag291_Supplementary_Data

## Data Availability

All the data are made available in the form of supplementary data. The detailed protocol is available at Protocols.io (dx.doi.org/10.17504/protocols.io.36wgq1qrkvk5/v1).

## Supplementary data

The following supplementary data are available at *JXB* online.

Fig. S1. Comparison of measurement techniques and time of measurement for potassium spiking analysis.

Fig. S2. Comparison of time of measurement for sodium spiking with different concentrations.

Table S1. Composition of Hyponex nutrient solution used for *Arabidopsis thaliana* growth.

Table S2. Composition of Yoshida nutrient solution used for *Oryza sativa* growth.

Table S3. Composition of *Lactuca sativa* nutrient solution.

Table S4. Data used for correlation plots of sodium and potassium, comparing analysis techniques MXRF and ICP-MS.

Table S5. MXRF analysis of Na and Cl in NaCl-spiked samples.

Table S6. Lower limit of detection of different compounds.

Table S7. Comparison of isotope XRF (Fe-55 source) and polychromatic (Rh-anode), WD-XRF, and MXRF methods using Na.

Table S8. Comparison of polychromatic XRF with Fe-55 source and Rh anode, wavelength dispersive XRF, and monochromatic XRF methods using K and Cl.
