# Supplementary material for: Quantitative light element profiling in plant tissues with monochromatic X-ray fluorescence analysis: a new frontier for abiotic stress studies
Source: J Exp Bot. 2026 Jun 17;77(17):5545–59. doi: 10.1093/jxb/erag291 (PMC13577731; doi:10.1093/jxb/erag291)
Supplement: Supplementary file 1 [file erag291_supplementary_data.pdf]

Supplementary information

Quantitative light element profiling in plant tissues with monochromatic X-ray fluorescence analysis: a new frontier for abiotic stress studies

Parvinderdeep S. Kahlon[1*], Pinelopi Kokkinopoulou[1], Silvia Bugallo Alfageme[1], Cheuk Ka Leong[1], Chenyu Zhang[2], Zewu Chen[3], Christa Testerink[1*] & Antony van der Ent[2*]

[1]Laboratory of Plant Physiology, Wageningen University and Research, Wageningen, The Netherlands.

[2]Laboratory of Genetics, Wageningen University and Research, Wageningen, The Netherlands.

[3]Z-Spec, New York, United States of America.

[*]Authors for correspondence: parvinderdeep.kahlon@wur.nl, christa.testerink@wur.nl & antony.vanderent@wur.nl

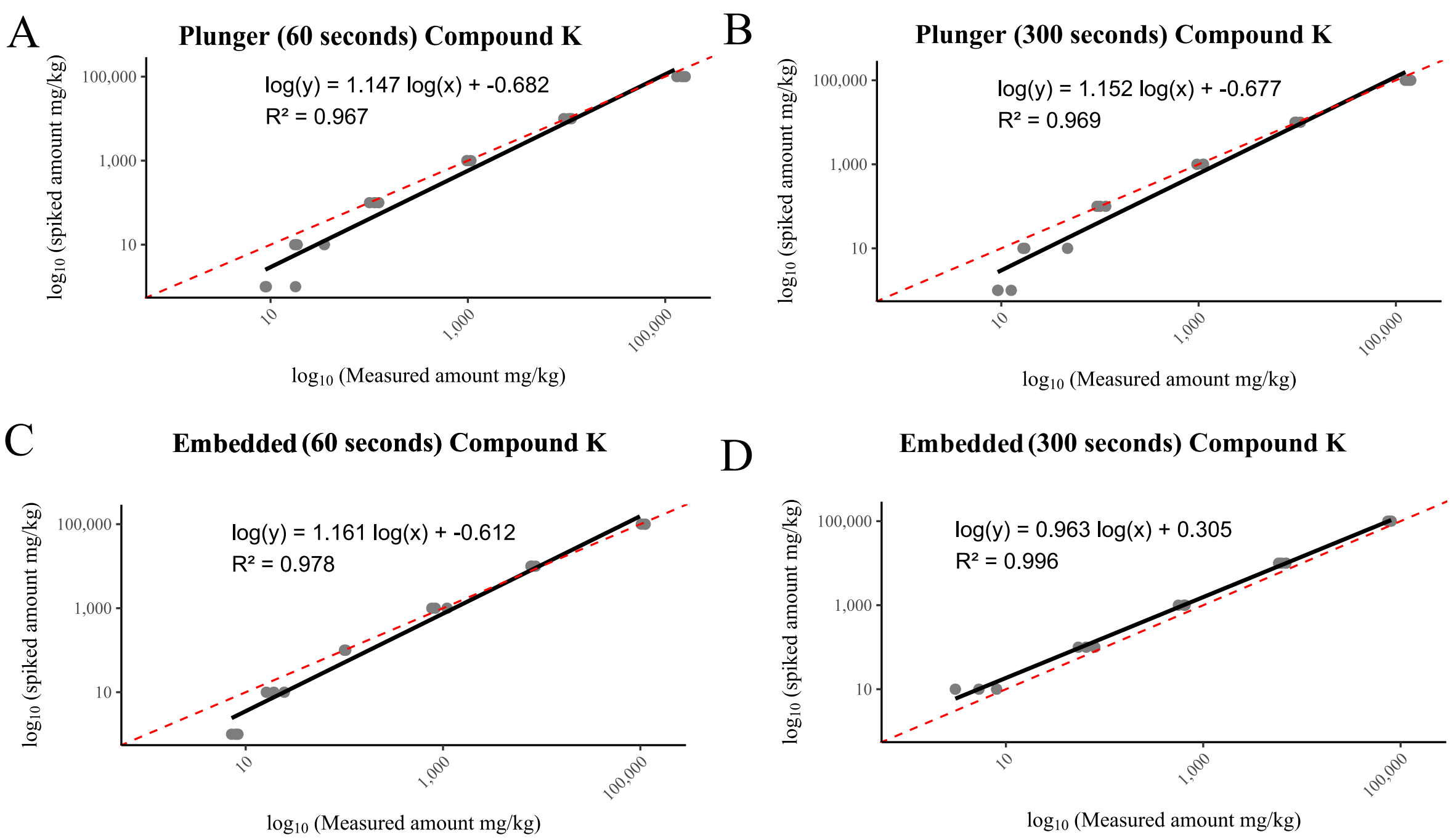

Supplementary Figure S1. Comparison of measurement techniques and time of measurement for potassium (K) spiking analysis using monochromatic X-ray fluorescence (XRF): (A) plunger method (60 s), (B) plunger method (300 s), (C) embedded method (60 s), and (D) embedded method (300 s). Data points represent K-spiked samples with different concentrations (1, 10, 100, 1000, 10,000, 100,000 mg/kg). Axes are displayed on a $\log_{10}$ scale. Pearson correlation coefficients (*r*) calculated from $\log_{10}$-transformed data are shown, indicating the strength and direction of the linear relationships for each method. Samples with K readout values below the instrument detection limit were not displayed on the $\log_{10}$-scaled figure.

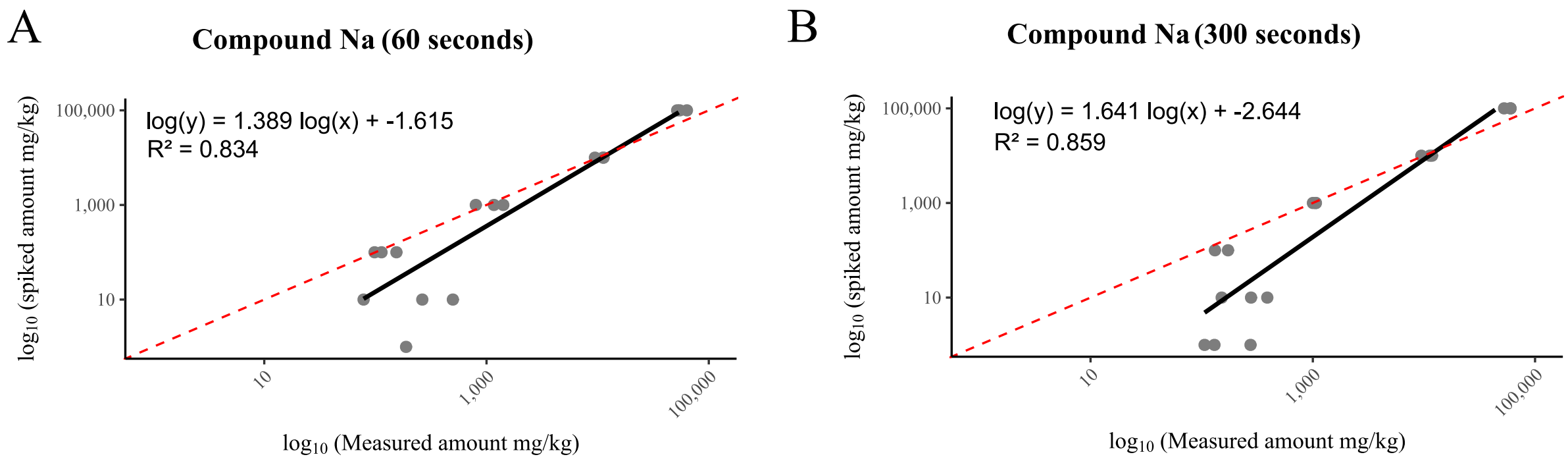

Supplementary Figure S2. Comparison of time of measurement for sodium (Na) spiking different concentrations (1, 10, 100, 1,000, 10,000, and 100,000 mg/kg) using sodium carbonate, with the embedded method (10 mg sample mass) and a measurement time of 60 s: (A) and 300 s: (B). Axes are displayed on a $\log_{10}$ scale. Pearson correlation coefficients (r) calculated from $\log_{10}$-transformed data are shown to indicate the strength and direction of the linear relationships between datasets. Samples with Na readout values below the instrument detection limit were not displayed on the $\log_{10}$-scaled figure.

Supplementary Table S1: Composition of Hyponex nutrient solution used for *Arabidopsis thaliana* growth (provided by UniFarm, Wageningen University and Research, The Netherlands).

**Macronutrients**	**Working concentration (mM)**
NH_4^+	1.7
K^+	4.13
Ca_2^+	1.97
Mg_2^+	1.24
NO_3^-	4.14
SO_42^-	3.14
$H2PO_4^-$	1.29
**Micronutrients**	**Working concentration (µM)**
Mn	3.4
Zn	4.7
B	14
Cu	6.9
Mo	0.5
Fe	21

Supplementary Table S2: Composition of Yoshida nutrient solution used for *Oryza sativa* (rice) growth (Yoshida et al., 1976).

**Macronutrients**	**Source**	**Working concentration (mM)**
Stock 1	NH_4NO_3	1.43
Stock 2	$CaCl_2.6H_2O$	1
Stock 3	$NaH_2PO_4.H_2O$	0.32
	K_2SO_4	0.51
	$MgSO_4.7H_2O$	1.64
**Stock 4 Micronutrients**	**Source**	**Working concentration (µM)**
Fe	$FeCl_3.6H_2O$	59.34
	Citric acid (pure)	77.42
B	H_3BO_3	18.88
Mn	$MnCl_2.4H_2O$	9.47
Zn	$ZnSO_4.7H_2O$	0.15
Cu	$CuSO_4.5H_2O$	0.24
Mo	$(NH_4)_6Mo_7O_{24}.4H_2O$	0.07

Supplementary Table S3: Composition of lettuce nutrient solution used for *Lactuca sativa* (lettuce) growth (Ciriello et al., 2021).

**Macronutrients**	**Working concentration (mM)**
$N\text{-}NO_3$	8.2
$N\text{-}NH_4$	1
P	0.7
K	2.7
Ca	2.9
Mg	0.7
Na	0
$S\text{-}SO_4$	1
**Micronutrients**	**Working concentration (µM)**
Cl	0
Fe	20
B	20
Cu	0.3
Zn	1.6
Mn	9
Mo	0.3

Supplementary Table S4: Data used for correlation plots of sodium (Na) and potassium (K), comparing analysis techniques Monochromatic X-ray Fluorescence (XRF), and ICP-MS across different plant tissues.

Sr. No.	Method	Plant	Tissue	Treatment (NaCl)	Replicate	ICP-MS (mg/kg)		XRF (mg/kg)	
						Na	K	Na	K
1	Plunger	*Arabidopsis thaliana*	Shoot	0 mM	1	3170	35400	4610	36200
2	Plunger	*Arabidopsis thaliana*	Shoot	0 mM	2	3780	35900	4750	28500
3	Plunger	*Arabidopsis thaliana*	Shoot	0 mM	3	3680	36200	4220	30000
4	Plunger	*Arabidopsis thaliana*	Shoot	0 mM	4	3430	34400	4330	28600
5	Plunger	*Arabidopsis thaliana*	Shoot	75 mM	1	26900	35900	17800	16800
6	Plunger	*Arabidopsis thaliana*	Shoot	75 mM	2	14100	17500	19300	16600
7	Plunger	*Arabidopsis thaliana*	Shoot	75 mM	3	13300	19500	15600	16100
8	Plunger	*Arabidopsis thaliana*	Shoot	75 mM	4	12500	16300	17100	13600
9	Plunger	*Arabidopsis thaliana*	Shoot	100 mM	1	17900	12500	20500	11600
10	Plunger	*Arabidopsis thaliana*	Shoot	100 mM	2	18500	13600	21500	11700
11	Plunger	*Arabidopsis thaliana*	Shoot	100 mM	3	18300	16000	19000	14000
12	Plunger	*Arabidopsis thaliana*	Shoot	100 mM	4	16400	14600	21100	12800
13	Plunger	*Arabidopsis thaliana*	Shoot	125 mM	1	25100	11100	25900	9880
14	Plunger	*Arabidopsis thaliana*	Shoot	125 mM	2	26100	10500	27000	10000
15	Plunger	*Arabidopsis thaliana*	Shoot	125 mM	3	20000	13800	23100	12700
16	Plunger	*Arabidopsis thaliana*	Shoot	125 mM	4	20300	13300	24900	12200
17	Plunger	*Arabidopsis thaliana*	Shoot	150 mM	1	27600	12500	25500	11400
18	Plunger	*Arabidopsis thaliana*	Shoot	150 mM	2	32800	12600	27900	10600
19	Plunger	*Arabidopsis thaliana*	Shoot	150 mM	3	29500	10500	19400	6340
20	Plunger	*Arabidopsis thaliana*	Shoot	150 mM	4	25800	11300	22600	8690
21	Plunger	*Lactuca sativa* (Salinas)	Shoot	0 mM	1	259	77000	4500	109000
22	Plunger	*Lactuca sativa* (Salinas)	Shoot	0 mM	2	289	84200	6400	132000
23	Plunger	*Lactuca sativa* (Salinas)	Shoot	0 mM	3	288	86000	3500	117000

24	Plunger	*Lactuca sativa* (Salinas)	Shoot	0 mM	4	297	86300	10400	130000
25	Plunger	*Lactuca sativa* (Salinas)	Shoot	0 mM	5	277	78800	6000	127000
26	Plunger	*Lactuca sativa* (Salinas)	Shoot	100 mM	1	34800	65200	39300	47800
27	Plunger	*Lactuca sativa* (Salinas)	Shoot	100 mM	2	35100	52400	38400	40200
28	Plunger	*Lactuca sativa* (Salinas)	Shoot	100 mM	3	36600	60200	49000	54100
29	Plunger	*Lactuca sativa* (Salinas)	Shoot	100 mM	4	35100	65100	39400	50800
30	Plunger	*Lactuca sativa* (Salinas)	Shoot	100 mM	5	34900	59500	41600	46000
31	Plunger	*Lactuca sativa* (Cobham)	Shoot	0 mM	1	377	84400	10600	127700
32	Plunger	*Lactuca sativa* (Cobham)	Shoot	0 mM	2	351	80800	9300	79200
33	Plunger	*Lactuca sativa* (Cobham)	Shoot	0 mM	3	346	81100	9900	110000
34	Plunger	*Lactuca sativa* (Cobham)	Shoot	0 mM	4	403	92000	9400	147000
35	Plunger	*Lactuca sativa* (Cobham)	Shoot	100 mM	1	41700	46700	40200	36700
36	Plunger	*Lactuca sativa* (Cobham)	Shoot	100 mM	2	49300	52200	43700	41900
37	Plunger	*Lactuca sativa* (Cobham)	Shoot	100 mM	3	48700	53200	39700	35600
38	Plunger	*Lactuca sativa* (Cobham)	Shoot	100 mM	4	42800	46900	41500	37300
39	Embedded	*Arabidopsis thaliana*	Shoot	0 mM	1	3170	35400	5100	30500
40	Embedded	*Arabidopsis thaliana*	Shoot	0 mM	2	3780	35900	4500	31200
41	Embedded	*Arabidopsis thaliana*	Shoot	0 mM	3	3680	36200	6300	29900
42	Embedded	*Arabidopsis thaliana*	Shoot	0 mM	4	3430	34400	6600	26600
43	Embedded	*Arabidopsis thaliana*	Shoot	75 mM	1	26900	35900	19500	15200
44	Embedded	*Arabidopsis thaliana*	Shoot	75 mM	2	14100	17500	20800	15800
45	Embedded	*Arabidopsis thaliana*	Shoot	75 mM	3	13300	19500	20300	16600
46	Embedded	*Arabidopsis thaliana*	Shoot	75 mM	4	12500	16300	20500	14800
47	Embedded	*Arabidopsis thaliana*	Shoot	100 mM	1	17900	12500	26100	11200
48	Embedded	*Arabidopsis thaliana*	Shoot	100 mM	2	18500	13600	25300	11800
49	Embedded	*Arabidopsis thaliana*	Shoot	100 mM	3	18300	16000	23500	14100
50	Embedded	*Arabidopsis thaliana*	Shoot	100 mM	4	16400	14600	25400	13700
51	Embedded	*Arabidopsis thaliana*	Shoot	125 mM	1	25100	11100	31400	9990
52	Embedded	*Arabidopsis thaliana*	Shoot	125 mM	2	26100	10500	34500	9540

53	Embedded	*Arabidopsis thaliana*	Shoot	125 mM	3	20000	13800	30200	12100
54	Embedded	*Arabidopsis thaliana*	Shoot	125 mM	4	20300	13300	29900	11500
55	Embedded	*Arabidopsis thaliana*	Shoot	150 mM	1	27600	12500	30700	10800
56	Embedded	*Arabidopsis thaliana*	Shoot	150 mM	2	32800	12600	35200	9790
57	Embedded	*Arabidopsis thaliana*	Shoot	150 mM	3	29500	10500	31100	9820
58	Embedded	*Arabidopsis thaliana*	Shoot	150 mM	4	25800	11300	31600	11000
59	Embedded	*Lactuca sativa* (Salinas)	Shoot	0 mM	1	259	77000	3540	82000
60	Embedded	*Lactuca sativa* (Salinas)	Shoot	0 mM	2	289	84200	2780	87500
61	Embedded	*Lactuca sativa* (Salinas)	Shoot	0 mM	3	288	86000	2620	90900
62	Embedded	*Lactuca sativa* (Salinas)	Shoot	0 mM	4	297	86300	1680	102000
63	Embedded	*Lactuca sativa* (Salinas)	Shoot	0 mM	5	277	78800	1040	92900
64	Embedded	*Lactuca sativa* (Salinas)	Shoot	100 mM	1	34800	65200	39700	39700
65	Embedded	*Lactuca sativa* (Salinas)	Shoot	100 mM	2	35100	52400	39000	34400
66	Embedded	*Lactuca sativa* (Salinas)	Shoot	100 mM	3	36600	60200	39800	41800
67	Embedded	*Lactuca sativa* (Salinas)	Shoot	100 mM	4	35100	65100	37600	44200
68	Embedded	*Lactuca sativa* (Salinas)	Shoot	100 mM	5	34900	59500	38300	40300
69	Embedded	*Lactuca sativa* (Cobham)	Shoot	0 mM	1	377	84400	2000	90300
70	Embedded	*Lactuca sativa* (Cobham)	Shoot	0 mM	2	351	80800	5810	91700
71	Embedded	*Lactuca sativa* (Cobham)	Shoot	0 mM	3	346	81100	4430	88000
72	Embedded	*Lactuca sativa* (Cobham)	Shoot	0 mM	4	403	92000	3050	90200
73	Embedded	*Lactuca sativa* (Cobham)	Shoot	100 mM	1	41700	46700	38400	31200
74	Embedded	*Lactuca sativa* (Cobham)	Shoot	100 mM	2	49300	52200	41800	36200
75	Embedded	*Lactuca sativa* (Cobham)	Shoot	100 mM	3	48700	53200	38000	32700
76	Embedded	*Lactuca sativa* (Cobham)	Shoot	100 mM	4	42800	46900	39500	35100
77	Embedded	*Oryza sativa* (Mudgo)	Root	0 mM	1	2730	21600	12900	25100
78	Embedded	*Oryza sativa* (Mudgo)	Shoot	0 mM	1	279	21400	4150	26400
79	Embedded	*Oryza sativa* (Mudgo)	Root	0 mM	2	2860	22400	10800	27800
80	Embedded	*Oryza sativa* (Mudgo)	Shoot	0 mM	2	352	22500	8140	26700
81	Embedded	*Oryza sativa* (Mudgo)	Root	0 mM	3	2880	20300	13300	22800

82	Embedded	*Oryza sativa* (Mudgo)	Shoot	0 mM	3	388	24300	4860	29300
83	Embedded	*Oryza sativa* (Mudgo)	Root	0 mM	4	2870	21200	14200	25300
84	Embedded	*Oryza sativa* (Mudgo)	Shoot	0 mM	4	8330	19100	9270	24800
85	Embedded	*Oryza sativa* (Mudgo)	Root	25 mM	1	17000	24200	22300	18000
86	Embedded	*Oryza sativa* (Mudgo)	Shoot	25 mM	1	8430	19000	18900	23100
87	Embedded	*Oryza sativa* (Mudgo)	Root	25 mM	2	14000	22200	20600	22100
88	Embedded	*Oryza sativa* (Mudgo)	Shoot	25 mM	2	6410	23200	19200	22900
89	Embedded	*Oryza sativa* (Mudgo)	Root	25 mM	3	14400	23700	21800	26400
90	Embedded	*Oryza sativa* (Mudgo)	Shoot	25 mM	3	13400	17200	21200	17900
91	Embedded	*Oryza sativa* (Mudgo)	Root	25 mM	4	15300	22800	31500	23900
92	Embedded	*Oryza sativa* (Mudgo)	Shoot	25 mM	4	25800	21500	50000	18800
93	Embedded	*Oryza sativa* (Mudgo)	Root	50 mM	1	26800	17600	38200	17900
94	Embedded	*Oryza sativa* (Mudgo)	Shoot	50 mM	1	15800	19500	20000	20400
95	Embedded	*Oryza sativa* (Mudgo)	Root	50 mM	2	28600	18900	38800	17800
96	Embedded	*Oryza sativa* (Mudgo)	Shoot	50 mM	2	7360	18900	13900	22000
97	Embedded	*Oryza sativa* (Mudgo)	Root	50 mM	3	21100	18600	31700	17900
98	Embedded	*Oryza sativa* (Mudgo)	Shoot	50 mM	3	15500	18200	22500	18500
99	Embedded	*Oryza sativa* (Mudgo)	Root	50 mM	4	22600	15500	47700	17800
100	Embedded	*Oryza sativa* (Mudgo)	Shoot	50 mM	4	347	34200	3510	37200
101	Embedded	*Oryza sativa* (M. Blatec)	Root	0 mM	1	3150	25300	14400	24000
102	Embedded	*Oryza sativa* (M. Blatec)	Shoot	0 mM	1	414	40500	5840	46400
103	Embedded	*Oryza sativa* (M. Blatec)	Root	0 mM	2	3030	25100	9540	23300
104	Embedded	*Oryza sativa* (M. Blatec)	Shoot	0 mM	2	384	32000	4230	39200
105	Embedded	*Oryza sativa* (M. Blatec)	Root	0 mM	3	2740	28100	10240	32100
106	Embedded	*Oryza sativa* (M. Blatec)	Shoot	0 mM	3	314	31200	4090	39900
107	Embedded	*Oryza sativa* (M. Blatec)	Root	0 mM	4	3540	19900	11900	22000
108	Embedded	*Oryza sativa* (M. Blatec)	Shoot	0 mM	4	61200	17200	74700	16200
109	Embedded	*Oryza sativa* (M. Blatec)	Root	25 mM	1	12500	19900	16100	17400
110	Embedded	*Oryza sativa* (M. Blatec)	Shoot	25 mM	1	41300	16500	39600	17500

111	Embedded	*Oryza sativa* (M. Blatec)	Root	25 mM	2	13100	17900	5390	3080
112	Embedded	*Oryza sativa* (M. Blatec)	Shoot	25 mM	2	9480	18700	18500	20700
113	Embedded	*Oryza sativa* (M. Blatec)	Root	25 mM	3	10600	20000	20000	24700
114	Embedded	*Oryza sativa* (M. Blatec)	Shoot	25 mM	3	5790	28800	14800	30600
115	Embedded	*Oryza sativa* (M. Blatec)	Root	25 mM	4	13500	19700	13200	8590
116	Embedded	*Oryza sativa* (M. Blatec)	Shoot	25 mM	4	14200	22600	24700	23600
117	Embedded	*Oryza sativa* (M. Blatec)	Root	50 mM	1	21300	17000	29200	16300
118	Embedded	*Oryza sativa* (M. Blatec)	Shoot	50 mM	1	12800	21000	19700	23800
119	Embedded	*Oryza sativa* (M. Blatec)	Root	50 mM	2	22200	17900	27300	14100
120	Embedded	*Oryza sativa* (M. Blatec)	Shoot	50 mM	2	41000	14300	44600	15000

Supplementary Table S5: Na and Cl spiking (with NaCl) with different concentrations (100. 1000 and 10000 mg/kg) using the embedded method (10 mg) with measurement time (60 seconds). measurement was performed using monochromatic X-ray fluorescence (XRF). Measurements were taken in triplicates and values are shown as mean ± standard deviation.

Compound	Spiked concentration (mg/kg)	XRF (mg/kg)
Na	100	0 ± 0
Na	1000	269 ± 467
Na	10000	12700 ± 6810
Cl	100	213 ± 31.6
Cl	1000	1020 ± 169
Cl	10000	9310 ± 1030

Supplementary Table S6: Lower limit of detection (LLD) of compounds calculated as described in Kadachi and Al-Eshaikhm, 2012.

**Elements**	**Background (counts)**	**Sensitivity (counts/ppm)**	**LOD (mg/kg)**
Na	227	0.142	318
Mg	1273	1.49	72
Al	678	4.84	16
Si	1206	13.15	7.9
P	1037	52.35	1.8
S	2041	110.4	1.2
Cl	7258	273	0.94
K	6523	662	0.37
Ca	3042	1292	0.13

Supplementary Table S7: Comparison of isotope XRF (Fe-55 source) and polychromatic (Rh-anode), WD-XRF and MXRF methods using Na (as NaCl) with different concentrations. Measurements were taken in triplicates and values are shown as mean ± standard deviation (±). n.d. denotes not detected. All values in mg/kg.

Matrix (Spiked compound)	Spiked Na	Isotope XRF (Fe-55 source)	Polychromatic XRF (Rh-anode)	WD-XRF	MXRF
Cellulose (Na)	0	n.d.	n.d.	n.d.	n.d.
Cellulose (Na)	1	n.d.	n.d.	n.d.	n.d.
Cellulose (Na)	10	n.d.	n.d.	n.d.	n.d.
Cellulose (Na)	100	n.d.	n.d.	n.d.	81.6 ± 110
Cellulose (Na)	1000	n.d.	n.d.	n.d.	774 ± 191
Cellulose (Na)	10000	n.d.	n.d.	9000 ± 266	12400 ± 2130
Cellulose (Na)	100000	n.d.	n.d.	36000 ± 3610	81000 ± 19300
NIST 1570a	Standard (18210 ± 230)	n.d.	n.d.	20900	18300
NIST 1567b	Standard (6.71 ± 0.21)	n.d.	n.d.	164.4	n.d.

Supplementary Table S8: Comparison of polychromatic XRF with Fe-55 source and Rh anode, wavelength dispersive XRF and monochromatic XRF methods using K and Cl (as NaCl and KCl) with different concentrations. Measurements were taken in triplicates. and values are shown as mean ± standard deviation. n.d. denotes not detected. All values in mg/kg.

Matrix (Spiked compound)	Spiked K	Spiked Cl	K				Cl			
			Isotope XRF (Fe-55 source)	Polychromatic XRF (Rh-anode)	WD-XRF	MXRF	Isotope XRF (Fe-55 source)	Polychromatic XRF (Rh-anode)	WD-XRF	MXRF
Cellulose (K + Cl)	n.d.	n.d.	n.d.	n.d.	n.d.	n.d.	n.d.	n.d.	n.d.	54.1 ± 10.9
Cellulose (K +Cl)	1	2.45	n.d.	n.d.	n.d.	n.d.	n.d.	n.d.	n.d.	61.9 ± 7.09
Cellulose (K +Cl)	10	24.5	8.1 ± 14.03	n.d.	n.d.	n.d.	23.4 ± 40.5	n.d.	n.d.	78.2 ± 11.3
Cellulose (K +Cl)	100	245	36.7 ± 63.50	52 ± 90	113 ± 2.21	71 ± 5.53	115 ± 199	6400 ± 11100	1200 ± 134	300 ± 36.5
Cellulose (K +Cl)	1000	2450	807 ± 699	1060 ± 956	1000 ± 41	827 ± 81.1	1840 ± 1590	1170 ± 1080	5870 ± 379	2650 ± 330
Cellulose (K +Cl)	10000	24500	2900 ± 5020	11100 ± 305	10100 ± 208	7590 ± 890	6970 ± 12100	27300 ± 6720	46200 ± 985	24300 ± 3190
Cellulose (K +Cl)	100000	245000	17100 ± 29600	77200 ± 12700	51100 ± 3060	53100 ± 12900	41000 ± 71000	181000 ± 30800	197000 ± 12800	185000 ± 49000
NIST 1570a	29000 ± 260	–	28600	28600	30000	29000	3350	3350	15900	8590
NIST 1567b	1325 ± 20	565.3 ± 8.6	753	753	1250	1080	571	571	1850	563
